# Analysis of gene expression array in TSC2-deficient AML cells reveals IRF7 as a pivotal factor in the Rheb/mTOR pathway

**DOI:** 10.1038/cddis.2014.502

**Published:** 2014-12-04

**Authors:** V Makovski, J Jacob-Hirsch, C Gefen-Dor, B Shai, M Ehrlich, G Rechavi, Y Kloog

**Affiliations:** 1 Department of Neurobiology, The George S Wise Faculty of Life Sciences, Tel-Aviv University, Tel-Aviv, Israel; 2 Department of Pediatric Hematology−Oncology, Safra Children's Hospital, Sheba Medical Center, Tel Hashomer, Israel; 3 Department of Cell Research and Immunology, The George S Wise Faculty of Life Sciences, Tel-Aviv University, Tel-Aviv, Israel; 4 Sackler Faculty of Medicine, Tel-Aviv University, Tel-Aviv, Israel

## Abstract

Mutations in tuberous sclerosis (*TSC*) genes cause the genetic disorder TSC, as well as other neoplasms, including lymphangioleiomyomatosis (LAM) and angiomyolipomas (AMLs). AMLs are benign renal tumors occur both in sporadic LAM and in TSC. As they carry the same mutations, AML cell lines serve as a model for TSC and LAM. Rheb/mammalian target of rapamycin complex 1 (mTORC1) pathway is chronically activated in TSC-deficient cells, and this activation can be diminished using the appropriate inhibitors. Rapamycin (sirolimus) is a known specific inhibitor of mTORC1, whereas S-trans,trans-farnesylthiosalicylic acid (FTS; salirasib) has been shown to inhibit Rheb. To examine the effect of the Rheb/mTOR inhibition pathway, we used human TSC2-deficient AML cells, derived from a LAM patient. FTS indeed inhibited Rheb in these cells and attenuated their proliferation. After comparative treatments with FTS or rapamycin or by re-expression of TSC2, we carried out a gene array analysis. This yielded a substantial number of commonly altered genes, many of which we identified as downstream targets of the interferon (IFN) regulatory factor 7 (IRF7) transcription factor, a central activator of the IFN type 1 immune response. Furthermore, nuclear localization of IRF7 was impaired by each of the three treatments. Interestingly, the phenomena seen on FTS or rapamycin treatment were selective for TSC2-deficient cells. Moreover, knockdown of IRF7 by siRNA mimicked the decrease in number of the abovementioned genes and also inhibited AML cell proliferation. Altogether, these findings support FTS as a potential treatment for TSC and its related pathologies and IRF7 as a novel target for treatment.

Tuberous sclerosis (TSC) is a genetic disease characterized by neurological disorders, as well as by benign tumors in various organs and is caused by a germline inactivating mutation in one of the *TSC* genes – *TSC1* or *TSC2*.^1^ About 40% of women with TSC will develop pulmonary lymphangioleiomyomatosis (LAM), which is a lethal disease affecting mainly women of childbearing age.^2^ It is characterized by proliferation and migration of smooth muscle cells from an unknown source into the lung, resulting in the obstruction of small airways and lymphatic vessels and leading to cystic parenchymal destruction and eventually death.^3, 4, 5^ There are two types of LAM: sporadic LAM (S-LAM) and LAM associated with TSC.^2^

About 80% of TSC patients and 40% of patients with S-LAM will develop renal angiomyolipomas (AMLs), benign kidney tumors containing fatty tissue, smooth muscle cells and dysplastic blood vessels.^6, 7, 8^ AML cells closely resemble LAM cells, as both carry inactivating mutations in either the *TSC1* or the *TSC2* gene.^7, 9, 10^ Previous studies raised the hypothesis that renal AML cells can generate pulmonary LAM by metastasizing into the lung.^7, 11^

The protein products of *TSC* genes act as GTPase-activating proteins for Rheb; consequently, loss of TSC1 or TSC2 expression leads to hyperactivation of the Rheb/mTOR pathway.^12^ Rheb belongs to the Ras superfamily of GTPases and terminates in a farnesylated CAAX motif.^13^ Our group has shown that activated Rheb-GTP is inhibited by farnesylthiosalicylic acid (FTS, salirasib) in TSC2-deficient cells of Eker rat leiomyoma (ELT3) cells.^14^ FTS, which was originally designed to mimic the farnesyl cysteine moiety of the COOH terminus of Ras, is currently undergoing clinical trials for cancer treatment.^15^ Rheb in its active form activates mammalian target of rapamycin complex 1 (mTORC1), a key regulator of protein translation, metabolism and cell proliferation.^16^ Rapamycin (sirolimus), a known inhibitor of mTORC1, has already been tested in LAM patients in the Multicenter International LAM Efficacy of Sirolimus (MILES) trial and has shown promising results that include improvement in lung function and alleviation of symptoms.^17^

Interferon (IFN) regulatory factors (IRFs) are a family of transcription factors that regulate the immune response to viral invasion by regulating IFN-induced immune response. They also have important roles in immune cell development, inflammation and oncogenesis.^18^ Mammalian cells harbor nine known members of the IRF family (IRF1–IRF9). IRF7, in conjunction with IRF3, is the main factor in regulation of the IFN type 1 response (IFN*α/β*). In its inactive state, IRF7 is localized in the cytoplasm. Following pathogen invasion it is activated by phosphorylation, and this leads to its translocation into the nucleus. As a transcription factor, IRF7 activates the transcription of IFN-stimulated genes, as well as small amounts of type 1 IFN (IFN*α/β*), and the latter leads to additional transcription of IRF7 via activation of the JAK/STAT signaling cascade. This positive feedback loop is essential for the elimination of viral invasion^19^ and also has antitumor and immunomodulatory functions.^20^ The Rheb/mTOR pathway was reported to participate in the activation of IRF7.^21^

In this study, we investigated the effect of FTS on human AML cells, which were shown to serve as a model for LAM.^7^ Using a gene array method, we demonstrate that FTS, by targeting Rheb, can inhibit the function of IRF7 in AML cells, thereby mimicking the effects of rapamycin and re-expression of TSC2.

## Results

### FTS and rapamycin inhibit proliferation of TSC2-null 621 cells by inhibiting the Rheb/mTORC1 signaling pathway

AMLs are benign kidney tumors containing smooth muscle cells, blood vessels and fat cells.^6^ These tumors are used to develop cell lines that can serve as models for LAM, as it is difficult to establish cell lines from pulmonary LAM cells.^7^ Here we used the AML cell line, 621, which is derived from a LAM patient and is deficient in TSC2.^7^ Having already shown that FTS inhibits the growth of TSC2-deficient rat ELT3 cells and inhibits Rheb but not Ras in those cells,^14^ we now wanted to substantiate those observations in a human model.

**Figure 1:**
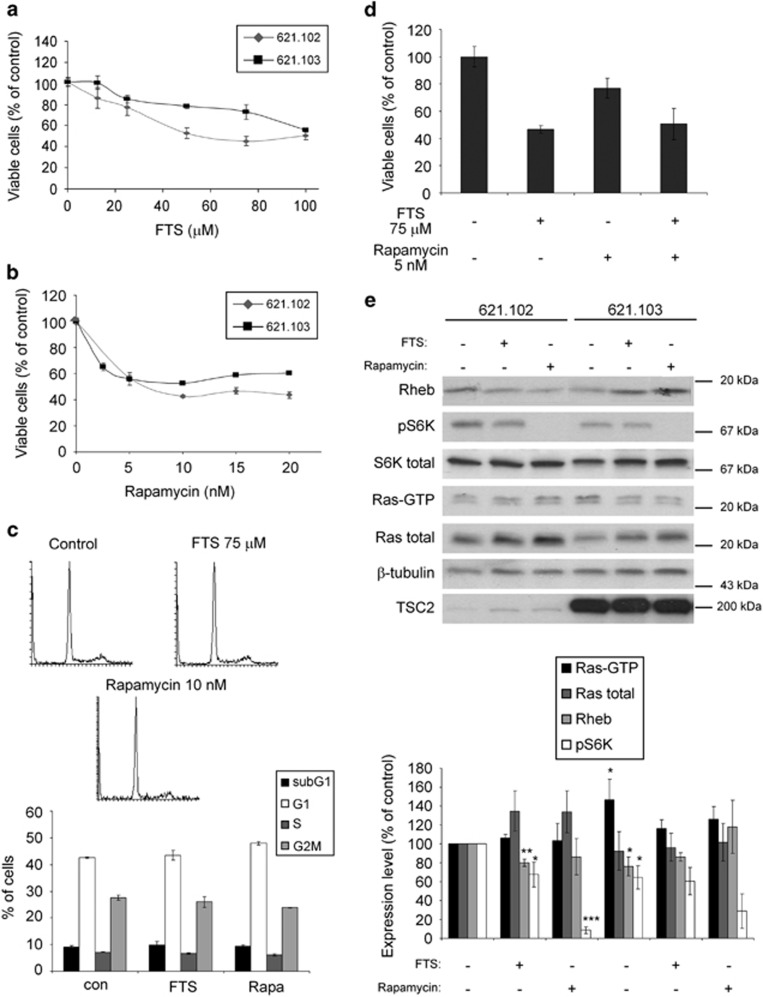
FTS inhibits AML cell proliferation and the Rheb/mTOR pathway, similarly to rapamycin. (**a**) The 621.102 (TSC2-deficient) and 621.103 (TSC2-re-expressing) AML cells were seeded and grown for 6 days in the absence and in the presence of the indicated concentrations of FTS, or with 0.1% Me_2_SO_4_ (control). Cells were directly counted and a typical inhibition curve is shown (means±S.E.M., *n*=3). (**b**) The 621.102 and 621.103 cells were seeded and grown for 6 days in the absence and in the presence of the indicated concentrations of rapamycin. Cells were directly counted and a typical inhibition curve is shown (means±S.E.M., *n*=3). (**c**) The 621.102 cells were seeded and grown for 2 days in the absence and in the presence of 75 *μ*M FTS or 10 nM rapamycin or 0.1% Me_2_SO_4_ (control). Cells then were fixed, PI stained and subjected to FACS for cell cycle analysis (see Materials and methods section). Typical histograms are shown in the upper panel. The quantification of the different cell cycle phases is shown in the lower panel. No significant difference was observed after FTS or rapamycin treatment. (**d**) The 621.102 cells were seeded and grown for 6 days in the absence and in the presence of the indicated concentration of both FTS and rapamycin, or with 0.1% Me_2_SO_4_ (control). Cells were directly counted and the percentages of live cells are presented. (**e**) TSC2-deficient 621.102 and TSC2-re-expressing 621.103 cells were treated for 2 days with 75 *μ*M FTS or 10 nM rapamycin or 0.1% Me_2_SO_4_ (control). Rheb, p-389 S6K, total S6K, Ras-GTP, Ras, *β*-tubulin and TSC2 were assayed by immunoblotting, as described in Materials and methods section. Statistical analyses of immunoblots from three experiments are shown in the lower panel (*n*=3, *P<0.05, ** P<0.01, *** P<0.001)

For this purpose, we used the TSC2-deficient 621.102 and the TSC2-re-expressing 621.103 cells, which are stably transfected with an empty vector and the TSC2 vector, respectively.^22^ First, we examined the impact of FTS on growth of the 621.102 and 621.103 cell lines. Cells were treated with the indicated doses of FTS for 6 days and were then counted (Figure 1a). We found that FTS inhibited the proliferation of 621.102 cells in a dose-dependent manner, with an average IC_50_ of 53±3.3 *μ*M (*n*=3). The 621.103 cells were less sensitive to FTS treatment with an average IC_50_ of 90±5.7 *μ*M (*n*=3), indicating that FTS is selective to the TSC2-deficient cells as shown previously.^14^ In an accompanying experiment, we treated 621.102 cells for 6 days with the mTORC1 inhibitor rapamycin (Figure 1b), which yielded an IC_50_ of 5.5±2.3 nM (*n*=3). Similar to their response to FTS, 621.103 TSC2-expressing cells were less sensitive to rapamycin with an average IC_50_ of 17.5±2.5 nM (*n*=3).

In order to show the mechanism of cell growth inhibition by the two drugs, we performed cell cycle analysis of the 621.102 cells after treatment with FTS or rapamycin (Figure 1c). The experiment showed that there was no significant difference in the percentage of subG1 population of the 621.102 cells after treatment with FTS or rapamycin, which means that neither of the drugs induces cell death. This was also strengthened by cell counting experiment with Trypan blue (data not shown). No difference was observed in the percentage of other cell cycle populations, indicating that both drugs do not cause cell cycle arrest in any particular stage.

We then performed an additional experiment using a combination of 75 *μ*M FTS and 5 nM rapamycin on 621.102 cells (Figure 1d). Although FTS alone and rapamycin alone inhibited cell growth by 53±3% and 23±7% respectively, their combined treatment yielded an inhibition of only 50±12% (*n=*4). This showed that neither drug has any additive or synergistic effect, supporting our previous observation that FTS inhibits Rheb and that it does so by acting through the same pathway as rapamycin.

Next, we wanted to confirm that, as we showed previously in rat ELT3 cells,^14^ the observed inhibition of growth by FTS was attributable to inhibition of Rheb and not Ras. The cells were seeded in 10-cm plates, treated with 75 *μ*M FTS or 10 nM rapamycin, and lysed 2 days later. Samples were then immunoblotted with the indicated antibodies to Ras, Rheb and the downstream Rheb target pS6K. As shown in Figure 1e, FTS downregulated Rheb and its downstream target S6K (p-T389) in the 621.102 cells to the levels of 80±4% and 67.5±13.2% of control, respectively (*n*=3, *P*<0.05). The levels of active Ras-GTP and total Ras, however, did not change significantly (106±4.1% and 135±21.5%, respectively; *n*=3, *P*>0.05; Figure 1e). These results are consistent with those we obtained in TSC2-deficient rat ELT3 cells.^14^ We also found here that rapamycin almost completely abolished the level of phosphorylated S6K, reducing it to 8.6±3.9% (*n*=3, *P*<0.05) but did not significantly change the levels of Rheb or Ras-GTP. Re-expression of TSC2 mimicked the effects of FTS, reducing the levels of Rheb and pS6K to 76.3±10% and 64.4±12.2% respectively (*n*=3, *P*<0.05). Interestingly, TSC2 re-expression raised the level of active Ras-GTP to 146.8±21.7% (*n*=3, *P*<0.05). FTS and rapamycin increased the level of Rheb expression in 621.103 cells, the reason for that may be the involvement of other compensation mechanisms after re-expression of TSC2.

It should be noted that FTS treatment was selective only for the 621.102 TSC2-deficient cells. In the 621.103 TSC2-re-expressing cells, the treatment did not change the level of either Rheb or pS6K as compared with the untreated cells. As to rapamycin, it was shown to inhibit the phosphorylation of S6K also in the 621.103 cells (Figure 1e). We assume that the reason for the difference between FTS and rapamycin is that rapamycin is very specific to mTORC1, whereas FTS inhibits all farnesylated GTPases, including Ras. When Rheb-GTP levels are high, the steady state is shifted toward Rheb inhibition. Upon TSC2 re-expression, Rheb-GTP levels decrease, whereas Ras-GTP is upregulated and the steady state is shifted toward Ras inhibition in this case. This is possibly the reason for the observed selective effect of FTS.

The fact that FTS can inhibit the growth of the AML cell line (which serves as an *in vitro* human model for LAM) and inhibits Rheb in these cells supports our suggestion that FTS should be considered as a possible treatment for LAM.

### Impact of FTS, rapamycin and TSC2 on gene expression in AML cells

**Figure 2:**
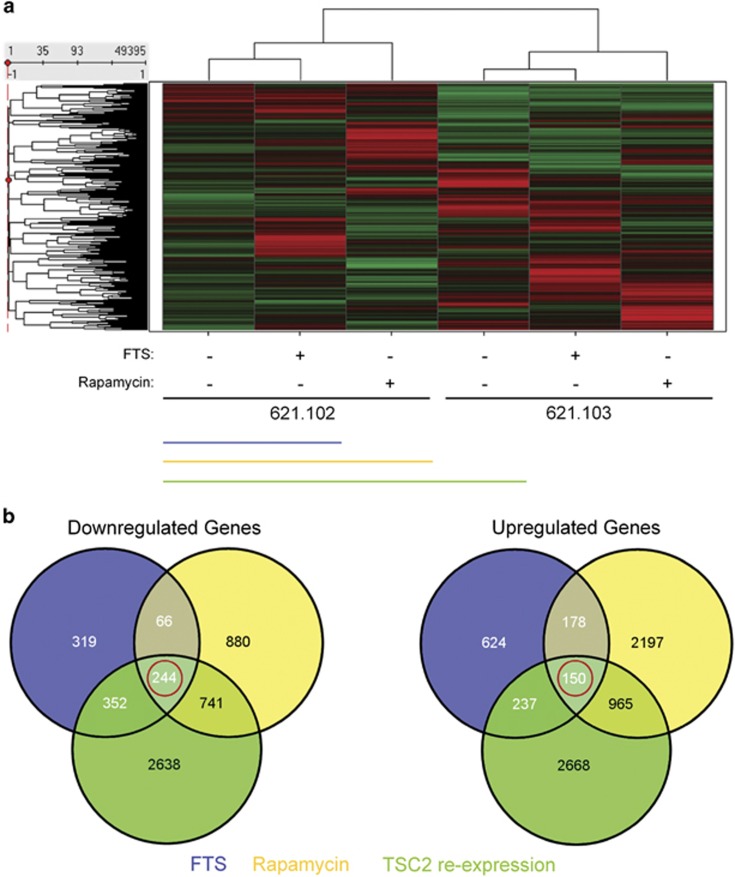
Comparative gene array analysis of 621.102 and 621.103 cells treated with FTS or rapamycin. (**a**) Hierarchical clustering of 49 395 probes, depicted in a dendrogram. Red, high relative expression; green, low relative expression. Genes are shown in columns; samples are shown in rows. (**b**) Venn diagram of the genes downregulated or upregulated by FTS *versus* control in 621.102 cells (blue), by rapamycin *versus* control in 621.102 cells (yellow) and by TSC2 re-expression in 621.103 control cells *versus* 621.102 control cells (green). The genes in red circles were analyzed further

Having now recapitulated the impact of FTS on Rheb in TSC2-deficient human cells (Figure 1), our next task was to compare the effects of FTS and rapamycin treatment and TSC2 re-expression on a larger scale. For this purpose, we performed a gene array analysis on the AML cell lines. We seeded 621.102 and 621.103 cells in 10-cm plates and treated them with 75 *μ*M FTS or 10 nM rapamycin for 48 h. As rapamycin is known to specifically inhibit mTORC1, which is a direct downstream target of Rheb,^23^ we used it as a positive control. We then resuspended the cells in TRIzol for RNA extraction and performed gene array profiling as described in Materials and methods section (Figure 2a). Using a 1.5-fold change in gene expression level as a criterion, we then analyzed only the group of genes that were either downregulated (244 genes; Figure 2b) or upregulated (150 genes) under the three conditions (FTS treatment, rapamycin treatment and TSC2 re-expression; Figure 2b). This was done to restrict the size of the group of analyzed genes to that related to the Rheb/mTOR pathway.

### FTS affects the expression of genes involved in the IFN type 1 immune response

**Figure 3:**
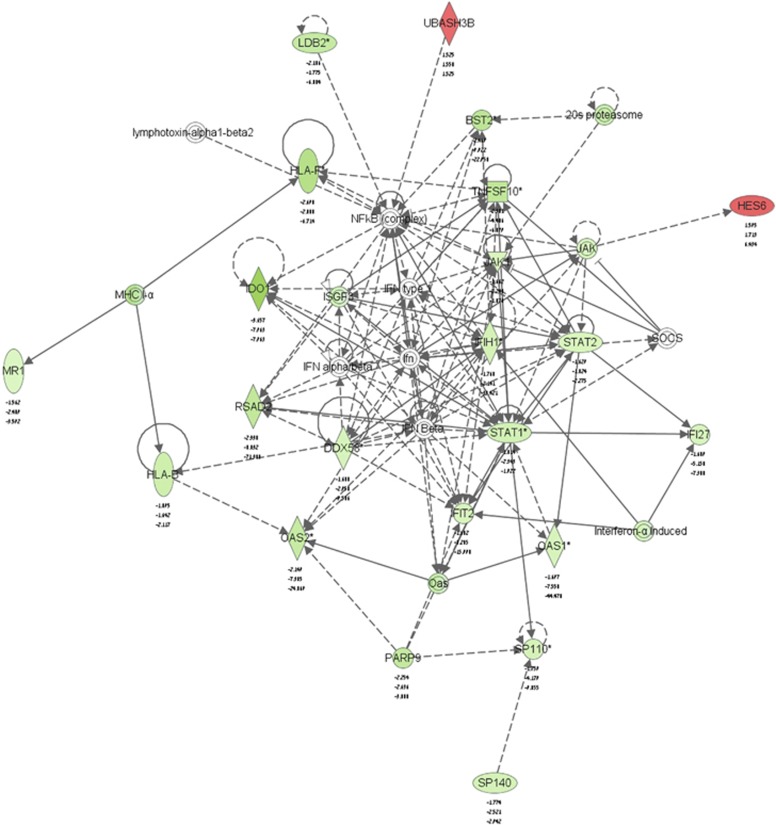
Network of the IFN type 1 pathway. Shown are fold decreases and increases in the expression of genes encoding the relevant enzymes relative to control (621.102 untreated cells) for each treatment. The network was produced using Ingenuity software

We used the DAVID Functional Annotation Clustering tool to analyze the common genes identified above. In the initial analysis, we found that the most prominent groups of genes were those associated with response to virus, regulation of cell death and defense response (Table 1). These results are unique when compared with a variety of gene expression profiles that we obtained previously, with and without FTS, in different cancer cells.^24, 25, 26^ FTS is shown here for the first time to affect genes involved in the immune response. Ingenuity software (QIAGEN, Redwood City, CA, USA) showed that a large proportion of the altered genes belong to the IFN type 1 signaling pathway (Figure 3).

**Figure 4:**
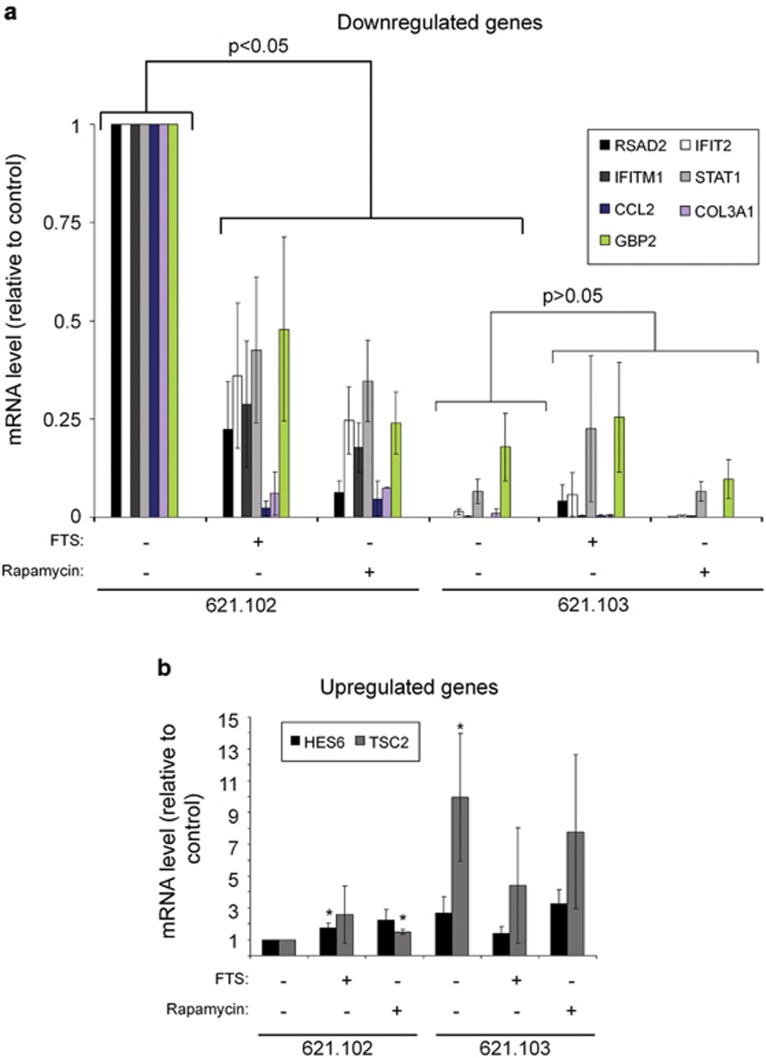
Validation of representative genes from the gene array using qRT-PCR. The 621.102 and 621.103 cells were treated for 48h with 75 *μ*M FTS, 10 nM rapamycin or 0.1% Me_2_SO_4_ (control). The mRNA levels of the indicated genes were then quantified with specific primers by RT-PCR (see Materials and methods section). Downregulated (**a**) and upregulated genes (**b**) are shown. The results correlate with the gene array analysis (means±S.E.M., *n*=3)

To validate the results of the gene array, we randomly chose nine genes from the abovementioned prominent groups with relatively high expression levels so as to make it easier to validate using qRT-PCR (Table 2). Cells were seeded and treated in the same way as for the gene array and were then subjected to quantitative RT-PCR with specific primers, as described in Materials and methods section. We found that the qRT-PCR results indeed correlated with the gene array results. Compared with 621.102, the expression levels of the indicated genes were downregulated (Figure 4a) or upregulated (Figure 4b) by treatment with FTS or rapamycin in the 621.102 cells and by TSC2 re-expression in the 621.103 cells, as anticipated from the gene array. We can see, as expected, the increase in TSC2 expression in the 621.103 cells (Figure 4b). Consistently with the gene array results, treatment with FTS or rapamycin did not affect expression levels in the 621.103 cells. These results indicated that both FTS and rapamycin treatment are selective only for the TSC2-deficient cells, a finding compatible with our results shown here in Figure 1d.

### FTS, rapamycin and re-expression of TSC2 inhibit the IRF7 transcription factor

**Figure 5:**
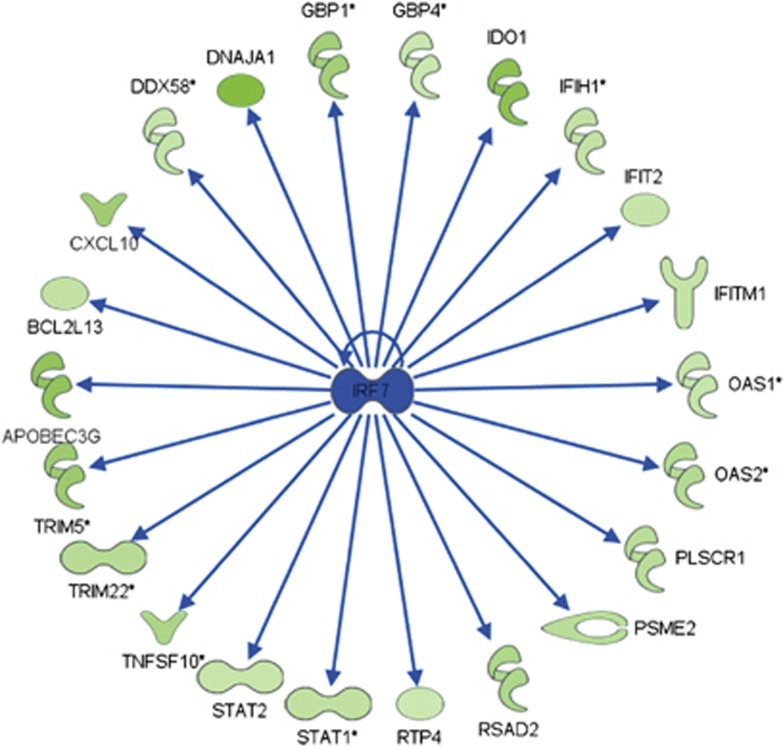
Scheme listing the downstream effectors of IRF7 that were downregulated in the gene array by FTS or rapamycin treatment or TSC2 re-expression. The scheme was produced using Ingenuity software

An 'upstream regulator' is a protein that is not itself altered in the gene array profiles, but many of its downstream targets are altered. Using the Ingenuity software, we obtained a list of upstream regulators, the most significant being the transcription factor IRF7 (Figure 5). We assayed the mRNA and protein levels of IRF7 under the different conditions (FTS or rapamycin treatment or TSC2 re-expression) using qRT-PCR and western immunoblotting (data not shown). We did not observe any substantial effects of FTS or rapamycin on the mRNA or protein levels of IRF7, which pointed to the involvement of some other mechanism of IRF7 inhibition.

**Figure 6:**
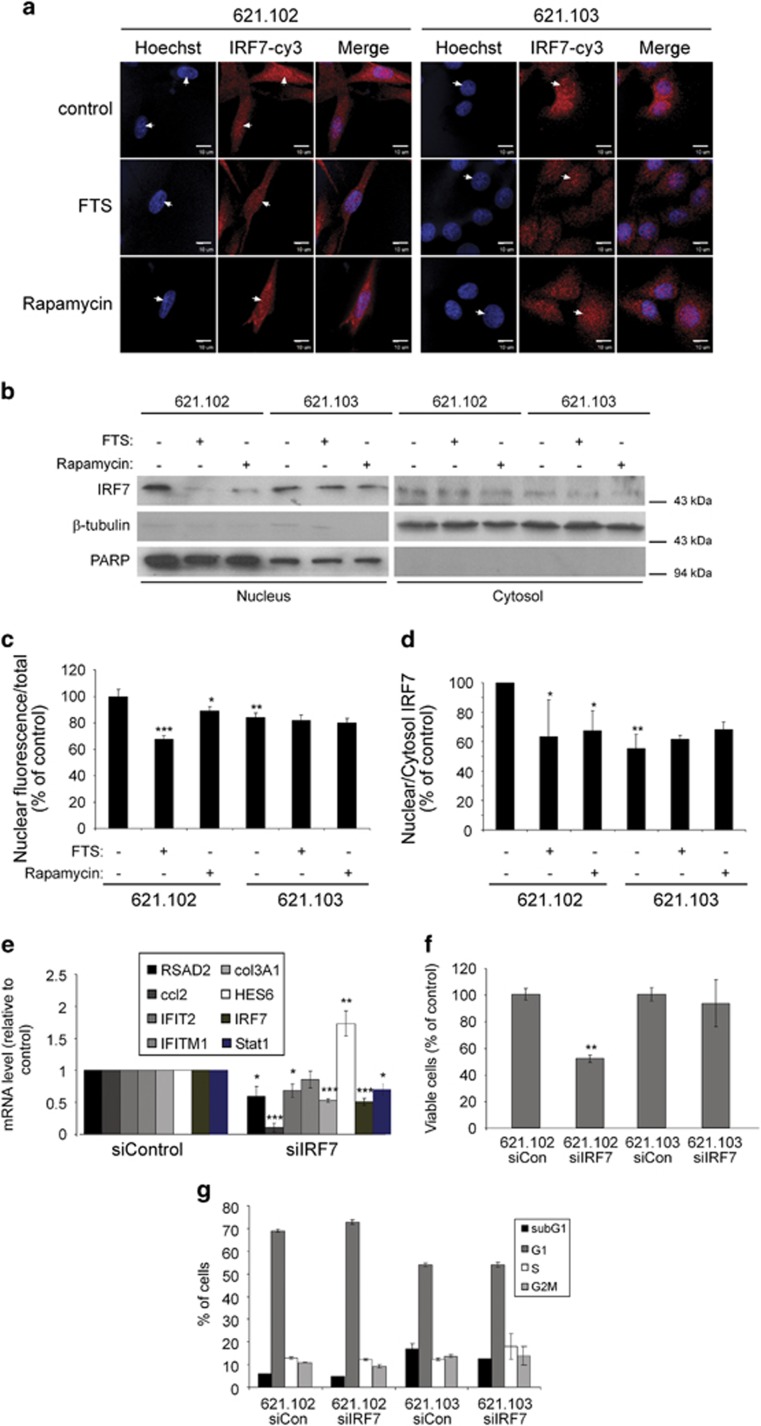
(**a**) FTS displaces the transcription factor IRF7 from the nucleus. The cells were treated similarly and then stained for confocal microscopy using anti-IRF7 Ab (see Materials and methods section). The nuclear localization of IRF7 was quantified using Hoechst staining of the nucleus (white arrow heads). Typical images of the cells are shown. (**b**) The cells were treated similarly and then subjected to nuclear fractionation (see Materials and methods section). IRF7 levels are shown in the nucleus and the cytosol. PARP and *β*-tubulin were used as nuclear and cytosolic markers, respectively, in order to show the quality of fraction separation. (**c**) Histograms of the IRF7 nuclear localization ratio (nuclear fluorescence/total cell fluorescence) as shown in **a**. FTS or rapamycin treatment or TSC2 re-expression decreased the nuclear fraction of IRF7 (means±S.E.M., *n*=30, *P<0.05, **P<0.01, ***P<0.001). Similar results were obtained from three separate experiments. (**d**) Histograms of the IRF7 nuclear localization ratio (nuclear/cytosol intensity) as shown in **b**. FTS or rapamycin treatment or TSC2 re-expression decreased the nuclear fraction of IRF7 (means±S.E.M., *n*=3, *P<0.05, **P<0.01). (**e**) Knockdown of IRF7 mimics the treatment with FTS or rapamycin or re-expression of TSC2. The 621.102 cells were transfected for 72 h with non-targeting control or IRF7 siRNA. The mRNAs of the indicated genes were then quantified with specific primers by qRT-PCR (see Materials and methods section). Their quantified levels are shown (means±S.E.M., *n*=3, *P<0.05, **P<0.01, ***P<0.001). (**f**) After transfection, 621.102 and 621.103 cells were re-seeded for 72 h and then counted to evaluate their proliferation rates. The percentage of cells relative to the control siRNA is shown. Downregulation of IRF7 reduced the proliferation rate of the 621.102 cells but did not change the proliferation rate of the 621.103 cells (means±S.E.M., *n*=4, **P<0.01). **(g)** After transfection, 621.102 and 621.103 cells were re-seeded for 48 h and then fixed, PI stained and subjected to FACS for cell cycle analysis (see Materials and methods section). The quantification of the different cell cycle phases is shown. No significant difference was observed after IRF7 knockdown

As IRF7 is a transcription factor that operates in the nucleus,^19^ we wanted to see whether FTS could displace IRF7 from the nucleus. Cells were seeded and treated as described in the previous experiment, and were then fixed with 4% PFA and stained with anti-IRF7 Ab (red fluorescence) or Hoechst (blue fluorescence) to label the nuclei (Figure 6a). Using confocal microscopy, we then quantified the proportion of the nuclear fraction of IRF7 inside the imaged cells (Figure 6c). The results showed that, compared with control, treatment with FTS or rapamycin or re-expression of TSC2 significantly decreased the nuclear fraction of IRF7 (to 67.5±2.8%, 88.8±3.6% or 84.2±3.2%, respectively, *P*<0.05, *n*=30; Figures 6a and 6c).

In addition, we also performed nuclear fractionation of 621.102 and 621.103 cell lysates. Cells were seeded and treated as described in the previous experiment, and were then lysed with cytosolic buffer and fractionated to nuclear and cytosolic fractions (see Materials and methods section). The fractions were subjected to western immunoblots with IRF7, *β*-tubulin and PARP antibodies. *β*-Tubulin was used as a cytosolic marker and PARP was used as a nuclear marker to show the quality of fraction separation (Figure 6b). Quantification of nuclear/cytosolic ratio of IRF7 showed a significant decrease in IRF7 nuclear localization after FTS or rapamycin treatment or TSC2 re-expression (to 67.5±2.8%, 88.8±3.6% or 84.2±3.2%, respectively, *P*<0.05, *n*=3; Figure 6d).

### Knockdown of IRF7 mimics the effects of Rheb/mTORC1 inhibition

To further establish IRF7 as an important factor in AML cell function, we used siRNA against IRF7 to knockdown its expression in 621.102 cells. First, we wanted to see whether inhibition of IRF7 could mimic the change in gene expression found in the gene array (Figure 4). For that purpose, 621.102 cells were seeded in six-well plates and transfected the next day with control siRNA or siRNA against IRF7. After 72h, the mRNA levels of the indicated genes were evaluated using qRT-PCR, as described in Materials and methods section (Figure 6e). The results showed that knockdown of IRF7 has a similar effect on the gene expression as FTS or rapamycin treatment or TSC2 re-expression (Figure 6e).

In addition, 621.102 and 621.103 cells transfected with control siRNA or siRNA against IRF7 were re-seeded in 24-well plates and then counted after 3 days to assess the rate of cell growth after IRF7 knockdown. As shown in Figure 6f, cells transfected with siRNA against IRF7 proliferated significantly more slowly (by 48±4.3% in 621.102 cells) than the cells transfected with non-targeting control siRNA (*n*=4*, P*<0.05; Figure 6f). Interestingly, siIRF7 knockdown had almost no effect on 621.103 cell proliferation, which strengthens the hypothesis that the observed effects of FTS, rapamycin and TSC2-re-epression are mediated by repression of IRF7 activity (Figure 6f).

To test the effect of IRF7 knockdown on cell cycle, we performed a cell cycle profile on the 621.102 and 621.103 cells after IRF7 knockdown. As shown in Figure 6g, there were no significant differences in cell cycle profile indicating that knockdown of IRF7 mimics the effect of FTS and rapamycin on cell cycle (Figure 1c).

Altogether, these accumulating data strongly suggest IRF7 is a pivotal factor in Rheb/mTOR pathway, and moreover that the inhibition of IRF7 activity is crucial for rapamycin or FTS-mediated effect. This strongly supports the contention that IRF7 is a target in LAM and other TSC-deficiency-related pathologies, such as TSC and cancer.

## Discussion

The results of this study strongly support our previous report^14^ that FTS inhibits the growth of TSC2-deficient rat ELT3 cells by affecting the active (GTP-bound) Rheb. In the present work, we showed that FTS can inhibit the growth not only of a rat cell line but also of a human TSC2-deficient AML cell line, which was derived from a LAM patient and can serve as a model for LAM^27^ (Figure 1a). This growth inhibition was similar to that induced by rapamycin (Figure 1b), since combined treatment with FTS and rapamycin yielded no additional effect beyond the effect seen by each drug alone (Figure 1d). Both drugs were selective for the TSC2-deficient cells (Figures 1a and 1b). FTS treatment decreased the levels of Rheb protein, as well as phosphorylation of its downstream target S6K (Figure 1e). As we showed previously,^14^ FTS downregulates the level of Rheb protein by inhibiting active Rheb-GTP and reducing Rheb stability. As the major part of Rheb protein is in its GTP-bound active form, we can assume that the reduction in Rheb protein levels in the 621 AML cells is also a result of Rheb-GTP inhibition.

We found a connection here between Rheb/mTORC1 inhibition and the IFN type 1 signaling pathway (Figure 3). Using microarray gene expression profiling of TSC2-deficient 621 AML cells, we looked specifically at overlapping genes that are altered by treatment of FTS or rapamycin or by TSC2 re-expression (Figure 2). We found that 244 and 150 genes were commonly downregulated and upregulated, respectively. Analysis of those genes showed significant enrichment of downstream targets of the IRF7 transcription factor (Figure 5), a key factor in IFN type 1-induced responses. Interestingly, the genes that were altered by FTS and rapamycin in the TSC2-deficient 621.102 AML cells were not affected by those treatments in the TSC2-re-expressing 621.103 cells, indicating that the treatments were selective for the TSC2-deficient cells only (Figure 4). This suggested that inhibition of cell growth by FTS or rapamycin is dependent on activation of Rheb/mTORC1.

TSC2/mTORC1 has been shown to have an important role in the generation of IFN-induced responses.^28^ Moreover, gene array analysis conducted previously on TSC complex cortical tubers, also revealed a connection between mutations in *TSC* genes and inflammation.^29^ It showed elevation of inflammatory gene expression in the tumor tissue, including *CCL2* and *GBP1*, which were also seen elevated in our study in AML cells. Other studies showed elevated levels of Stat1 in AML and LAM tissues.^30^ The exact mechanism leading to IFN type 1 activation is not yet known. In this study, we showed for the first time that inhibition of the Rheb/mTORC1 pathway results in a reduction in IRF7 nuclear localization (Figures 6a and 6c), which in turn decreases the transcription of IFN-stimulated genes (Figure 6e). Moreover, knockdown of IRF7 by siRNA leads to inhibition of the growth of TSC2-deficient AML cells (Figure 6f), indicating that IRF7 may be an important therapeutic target in the treatment of LAM and other TSC-related pathologies. Previous gene expression analyses have shown that FTS can downregulate the transcription factor HIF-1*α*, resembling the downregulation of IRF7 in this study.^25^

Studies have shown that TSC2-deficient cells have a reduced sensitivity to the growth inhibitory effects of IFN-*β* and that re-expression of TSC2 restores the anti-proliferative properties of this cytokine.^31^ Our results may explain this phenomenon, as we show here that the IFN type 1 response is heightened in TSC2-deficient AML cells independently of IFN-*β* expression. Inhibition of the Rheb/mTOR pathway leads to reduction in IRF7 and in the IFN type 1 immune response, which may repair the cellular response to IFN-*β*.

Treatments currently available for LAM are poor.^32^ The drug leading the field in clinical trials is rapamycin (sirolimus). The problem is that prolonged treatment with rapamycin may lead to a series of adverse effects. When rapamycin treatment is discontinued, patients continue to decline.^17^ As this work shows that the therapeutic activity of FTS is similar to that of rapamycin, the non-toxic properties of FTS make it beneficial for the treatment of LAM.^15^

Studies have shown that IFN-*γ* can inhibit the growth of AML lesions and that combined treatment with IFN-*γ* and rapamycin yields synergistic effects.^33^ In light of our new results presented here, it will be interesting to test a treatment combination of FTS with IFN-*γ*.

## Materials and Methods

### Cell culture and reagents

The TSC2-null 621.101 AML cell line was derived from a kidney of a LAM patient. The 621.102 and 621.103 cell lines were stably transfected with an empty vector and TSC2 vector, respectively.^27^ Both cell lines were kindly provided by Dr. DJ Kwiatkowski, Brigham and Women's Hospital, Harvard Medical School, Boston, MA, USA. The cells were maintained in high-glucose DMEM medium containing 10% FBS, 1% penicillin/streptomycin and 1% L-glutamine (Biological Industries, Beit HaEmek, Israel). The cells were incubated at 37 °C in a humidified atmosphere of 95% air and 5% CO_2_. FTS was a gift from Concordia Pharmaceuticals (New York City, NY, USA). Rapamycin was purchased from Sigma-Aldrich (Rehovot, Israel).

### Fluorescence-activated cell sorting analysis

In all, 4 × 10^5^ cells were plated per 10-cm dish. The following day, FTS 75 *μ*M, rapamycin 10 nM or 0.1% Me_2_SO_4_ (control) were added and cells were incubated for 48 h. Cells were then collected and resuspended with PBS containing propidium iodide (50 *μ*g/ml; Sigma, Rehovot, Israel) and 0.05% Triton X-100 (BDH, Arlington Heights, IL, USA) for DNA staining, then analyzed with a fluorescence-activated cell sorter (FACS Caliber; Becton Dickinson, Franklin Lakes, NJ, USA). Analysis was performed using Flowing Software (Turun Yliopisto, Finland).

### Western immunoblot analysis

The 621.102 and 621.103 cells were plated at density of 4 × 10^5^ cells per 10-cm plate, grown for 24 h, and then treated with FTS, rapamycin or 0.1% Me_2_SO_4_ (control) for 48h. The treated cells were lysed as described previously ^34^ and the lysates (100 *μ*g protein) were immunoblotted with mouse anti-pan-Ras Ab (Calbiochem, San Diego, CA, USA), rabbit anti-Rheb Ab (Cell Signaling, Danvers, MA, USA), rabbit anti-*β* tubulin Ab (Santa Cruz Biotechnology, Santa Cruz, CA, USA), rabbit anti-pS6K Ab, rabbit anti-S6K Ab (Sigma-Aldrich) and rabbit anti-IRF7 Ab (Abcam, Cambridge, UK). Immunoblots were exposed to the appropriate secondary peroxidase-coupled IgG (1 : 2500 dilution, Jackson ImmunoResearch Laboratories, West Grove, PA, USA) and subjected to enhanced chemiluminescence (Amersham Pharmacia Biotech, Piscataway, NJ, USA). Protein bands were quantified by densitometry with Image EZQuant-Gel Statistical Analysis Software.

### GTPase pull-down assay

Lysates containing 500 *μ*g protein were used to determine Ras-GTP content by the glutathione *S*-transferase Rho-binding domain pull-down assay, as described elsewhere.^35^

### Gene expression profiling

The effect of FTS or rapamycin on gene expression in 621.102 and 621.103 cells was determined 48 h after treatment with FTS (75 *μ*M), rapamycin (10 nM) or vehicle (a single microarray for each condition). The cells were lysed with TRIzol (Ambion Life Technologies, Grand Island, NY, USA) and analyzed using Affymetrix HU GENE1.0st oligonucleotide arrays (http://www.affymetrix.com/support/technical/datasheets/gene_1_0_st_datasheet.pdf).

Sample processing was performed according to the Affymetrix WT (Whole Transcript) protocol as described at (https://www.affymetrix.com/support/downloads/manuals/wt_sensetarget_label_manual.pdf).

### Analysis of gene expression data

Gene level RMA sketch algorithm (Affymetrix Expression Console, Santa Clara, CA, USA and Partek Genomics Suite 6.2, St. Louis, MO, USA) was used to generate crude data. Genes were filtered and analyzed using fold change calculations and unsupervised hierarchical cluster analysis (Spotfire DecisionSite for Functional Genomics; Somerville, MA, USA). Further processing included functional analysis and over-representation calculations based on gene ontology and published data from the Database for Annotation (GO), Visualization and Integrated Discovery (DAVID) (http://david.abcc.ncifcrf.gov/summary.jsp) and Ingenuity Software. Data results were deposited in http://eng.sheba.co.il/genomics.

### Total RNA purification and real-time PCR analysis

Total RNA was isolated using the PerfectPure RNA Cultured Cell Kit (5 Prime, Hilden, Germany) and then reverse transcribed and subjected to qRT-PCR as described previously.^14^ The primers used were from Real Time Primers, Elkins Park, PA, USA: human *CCL2* forward, 5′-GTGTCCCAAAGAAGCTGTG-3′ human *CCL2* reverse, 5′-GATTCTTGGGTTGTGGAGTG-3′ human *Col3A1* forward, 5′-AGCTACGGCAATCCTGAACT-3′ human *Col3A1* reverse, 5′-GGGCCTTCTTTACATTTCCA-3′ human *STAT1* forward, 5′-GCAAAACCTTGCAGAACAGA-3′ human *STAT1* reverse, 5′-ATCAGGGCATTCTGGGTAAG-3′ human *RSAD2* forward, 5′-TCTGAAGCGAGGAGGAAAAT-3′ human *RSAD2* reverse, 5′-GTTTTCAGCCACTGGGAAAT-3′ human *GBP2* forward, 5′-TTTCACCCTGGAACTGGAAG-3′ human *GBP2* reverse, 5′-GACGAAGCACTTCCTCTTGG-3′ human *IFIT2* forward, 5′-TGGAGGAAACCAAAATGAAA-3′ human *IFIT2* reverse, 5′-TCCTCTTCACCTTCTTCACG-3′ human *IFITM1* forward, 5′-AAAGCCAGAAGATGCACAAG-3′ human *IFITM1* reverse, 5′-GGAGTAGGCGAATGCTATGA-3′ human *TSC2* forward, 5′-GAAGTCGCAAAAACCAAGAA-3′ human *TSC2* reverse, 5′-TGTGTCTCCCATTGTCTGTG-3′ human *HES6* forward, 5′-CTACGGGCAGGAGGAAGAAT-3′ human *HES6* reverse, 5′-AGTGCACCTGCCTCTCATCT-3′ human *GAPDH* forward, 5′-CCAGAACATCATCCCTGC-3′ human *GAPDH* reverse, 5′-GGAAGGCCATGCCAGTGAGC-3′.

The relative mRNA expression of the target gene was normalized to the expression of the *glyceraldehyde 3-phosphate dehydrogenase* (*GAPDH*) reference gene.

### Confocal microscopy

The 621.102 and 621.103 cells (1 × 10^5^ of each) were plated on glass coverslips and treated for 48 h with 75 *μ*M FTS, 10 nM rapamycin or the vehicle (control; 0.1% Me_2_SO_4_). Cells were fixed and permeabilized as described previously.^36^ Cells were labeled for 1 h with 5 *μ*g/ml rabbit anti-IRF7 Ab (Abcam) and then with 1 : 750 goat anti-rabbit cy3 Ab (Jackson ImmunoResearch Laboratories). The slides were incubated with Hoechst 33258 (Sigma-Aldrich) for 10 min. Images were acquired with a Zeiss LSM 510 META confocal microscope or spinning-disk confocal microscope (CSU-22 confocal head (Yokogawa, Tokyo, Japan) and Axiovert 200 M instrument (Carl Zeiss MicroImaging, Jena, Germany)) under the control of the SlideBook program (Intelligent Imaging Innovations, Denver, CO, USA), using a 63 × oil immersion objective (Plan Apochromat; numerical aperture (NA), 1.4, Zeiss, Jena, Germany) and an Evolve electron-multiplying charge-coupled-device (EMCCD) camera (Photometrics, Tucson, AZ, USA). Fluorescence intensity was quantified by ImageJ software (NIH, Bethesda, MD, USA).

### Nuclear fractionation

The 621.102 and 621.103 cells were plated at density of 4 × 10^5^ cells per 10-cm plate, grown for 24 h, and then treated with FTS, rapamycin or 0.1% Me_2_SO_4_ (control) for 48 h. The treated cells were then lysed with cytosolic buffer (Hepes 10 mM, MgCl_2_ 1.5 mM, KCl 10 mM, IGEPAL 0.1%, DTT 0.5 mM and Proteinase inhibitor cocktail) and centrifuged at 960 *g* for 10 min. The sup (cytosol) was subjected to western immunoblot. The pellet (nuclei) was washed with cytosolic buffer, resuspended with the same buffer volume as the sup and subjected to western immunoblot.

### Transfection and siRNA

The 621.102 and 621.103 cells (2 × 10^5^) were plated in six-well plates and transfected after 24 h with 25 nM ON-TARGETplus IRF7 siRNA oligos, as well as ON-TARGETplus siCONTROL non-targeting pool (Thermo Scientific, Waltham, MA) using TransIT-siQUEST Transfection Reagent (Mirus, Madison, WI, USA) according to the manufacturer's instructions. As an indicator of transfected cells, we used the siGLO Green transfection indicator (Thermo Scientific). The cells were harvested after 72 h.

### Statistical analysis

Data are expressed as means±S.E.M.. Significant differences in mean values were assessed by Student's *t*-test. A value of *P*⩽0.05 was considered significant.

## Figures and Tables

**Table 1:** Biological processes most enriched in the analyzed genes

**Term**	**Count**	***%***	***P*-value**
GO:0009615 – response to virus	10	2.53	0.0009
GO:0010941 – regulation of cell death	34	8.61	0.0010
GO:0048518 – positive regulation of biological process	68	17.22	0.0010
GO:0006793 – phosphorus metabolic process	38	9.62	0.0016
GO:0006952 – defense response	27	6.84	0.0019
GO:0009611 – response to wounding	24	6.08	0.0025
GO:0048002 – antigen processing and antigen peptide presentation	5	1.27	0.0036
GO:0048522 – positive regulation of cellular process	60	15.19	0.0044
GO:0045087 – innate immune response	10	2.53	0.0045
GO:0051270 – regulation of cell motion	12	3.04	0.0049
GO:0019538 – protein metabolic process	84	21.27	0.0060
GO:0032879 – regulation of localization	25	6.33	0.0071
GO:0051272 – positive regulation of cell motion	8	2.03	0.0072
GO:0043069 – negative regulation of programmed cell death	17	4.30	0.0085
GO:0060548 – negative regulation of cell death	17	4.30	0.0088
GO:0040012 – regulation of locomotion	11	2.78	0.0129
GO:0030334 – regulation of cell migration	10	2.53	0.0158
GO:0051707 – response to other organism	14	3.54	0.0162
GO:0040017 – positive regulation of locomotion	7	1.77	0.0249
GO:0006897 – endocytosis	11	2.78	0.0300
GO:0010324 – membrane invagination	11	2.78	0.0300
GO:0006935 – chemotaxis	9	2.28	0.0308
GO:0042330 – taxis	9	2.28	0.0308
GO:0002682 – regulation of immune system process	16	4.05	0.0318
GO:0006082 – organic acid metabolic process	21	5.32	0.0330
GO:0001666 – response to hypoxia	8	2.03	0.0343
GO:0042180 – cellular ketone metabolic process	21	5.32	0.0363
GO:0042127 – regulation of cell proliferation	27	6.84	0.0381
GO:0010942 – positive regulation of cell death	17	4.30	0.0423
GO:0070482 – response to oxygen levels	8	2.03	0.0433
GO:0044093 – positive regulation of molecular function	21	5.32	0.0487

Ease score<0.05

**Table 2:** List of selected genes altered in the gene array

**Gene symbol**	**Gene title**	**Fold change**		
		**621.102 FTS *versus* 621.102 control**	**621.102 Rapa *versus* 621.102 control**	**621.103 Con *versus* 621.102 control**
*ADM*	Adrenomedullin	−1.5	−1.9	−4.3
*APOBEC3G*	Apolipoprotein B mRNA editing enzyme, catalytic polypeptide-like 3G	−3.4	−2.9	−19.7
*BST2*	Bone marrow stromal cell antigen 2	−2.5	−9.9	−23.0
*C3AR1*	Complement component 3a receptor 1	−2.0	−6.2	−8.6
*CASP1*	Caspase 1, apoptosis-related cysteine peptidase (interleukin 1, beta, convertase)	−1.9	−3.8	−3.3
*CCL2*^a^	Chemokine (C−C motif) ligand 2	−2.0	−1.6	−31.6
*COL14A1*	Collagen, type XIV, alpha 1	−2.5	−2.1	−19.3
*COL3A1*^a^	Collagen, type III, alpha 1	−1.6	−1.6	−54.9
*CPM*	Carboxypeptidase M	−3.5	−1.6	−2.4
*DDX58*	DEAD (Asp-Glu-Ala-Asp) box polypeptide 58	−1.5	−2.3	−13.2
*DRAM1*	DNA, damage-regulated autophagy modulator 1	−2.0	−1.9	−6.8
*GBP1*	Guanylate binding protein 1, interferon-inducible, 67 kDa	−2.7	−2.1	−8.2
*GBP2*^a^	Guanylate binding protein 2, interferon-inducible	−1.6	−4.2	−16.2
*HLA-F*	Major histocompatibility complex class I, F	−2.5	−2.1	−6.4
*HSPA5*	Heat shock 70-kDa protein 5 (glucose-regulated protein, 78 kDa)	−1.5	−6.5	−6.1
*ICAM1*	Intercellular adhesion molecule 1	−1.5	−1.6	−3.7
*IDO1*	Indoleamine 2,3-dioxygenase 1	−3.7	−8.0	−8.0
*IFI27*	Interferon, alpha-inducible protein 27	−1.7	−5.2	−7.4
*IFIH1*	Interferon induced with helicase C domain 1	−1.8	−2.1	−33.4
*IFIT2*^a^	Interferon-induced protein with tetratricopeptide repeats 2	−1.7	−8.2	−16.0
*IFITM1*^a^	Interferon-induced transmembrane protein 1 (9–27)	−1.7	−2.6	−128.5
*MMP3*	Matrix metallopeptidase 3 (stromelysin 1, progelatinase)	−1.7	−2.2	−8.1
*PLSCR1*	Phospholipid scramblase 1	−2.0	−2.6	−13.2
*RSAD2*	Radical S-adenosyl methionine domain-containing 2	−2.3	−8.8	−71.4
*STAT1*^a^	Signal transducer and activator of transcription 1, 91 kDa	−1.8	−4.5	−25.5
*TNFSF10*	Tumor necrosis factor (ligand) superfamily, member 10	−1.8	−5.5	−12.8
*TRIM22*	Tripartite motif-containing 22	−1.8	−1.9	−10.0
*TRIM5*	Tripartite motif-containing 5	−3.0	−2.1	−3.3
*TSC2*^a^	Tuberous sclerosis 2	1.5	1.6	2.9
*HES6*^a^	Hairy and enhancer of split 6 (*Drosophila*)	1.6	1.7	6.4
*RPL7*	Ribosomal protein L7	1.6	2.6	4.5

a Genes that were validated using qRT-PCR

## Abbreviations

TSC: tuberous sclerosis
LAM: lymphangioleiomyomatosis
AML: angiomyolipoma
FTS: farnesylthiosalicylic acid
IFN: interferon
IRF7: interferon regulatory factor 7
